# Effect of buckwheat bran protein enzymatic hydrolysates on the rheological, textural and structural properties of non-fermented wheat dough

**DOI:** 10.1016/j.fochx.2025.102501

**Published:** 2025-04-28

**Authors:** Libo Wang, Minghuan Yan, Qiaoming Jiang, Bing Wang, Denglin Luo, Chonghui Yue, Jinying Guo, Ju Qiu, Haoran Wang, Weijing Wu, Yilin Huang

**Affiliations:** a College of Food and Bioengineering, Henan University of Science and Technology, Luoyang, Henan 471023, China; b College of Food Science and Technology, Henan University of Technology, Zhengzhou, Henan, 450001, China; c Department of Nutrition and Health, China Agricultural University, No.17 Qinghuadonglu, Haidian, Beijing 100083, China; d College of Food Science and Engineering, Beijing University of Agriculture, Changping, Beijing 102206, China; e Xiamen Medical College, No.1999 Guankou Middle Road, Xiamen, Fujian, 361023, China

**Keywords:** Buckwheat bran, Protein hydrolysates, Rheological properties, Disulfide bonds, Amino acid composition, Microstructure

## Abstract

The effects of Tatary/common buckwheat bran protein enzymatic hydrolysates (TBBPEHs or CBBPEHs) on the physicochemical and structural properties of non-fermented wheat dough were investigated. The presence of TBBPEHs and CBBPEHs hydrolysates decreased the dough water absorption, storage modulus (*G'*), loss modulus (*G"*), hardness and chewiness, and increased the dough formation time. As the amount of substitution increased, the tightly bound water in the dough was gradually transformed into weakly bound water. The dough exhibited higher levels of *G'*, *G"* and disulfide bonds at 4 % substitution level compared to other complex doughs, suggesting that medium concentrations of hydrolysates could contribute to the stability of the gluten network. SEM and CLSM observations indicated that low levels of TBBPEHs and CBBPEHs hydrolysates (2–4 %) contributed to a homogeneous and more continuous gluten network structure. In addition, TBBPEHs and CBBPEHs hydrolysates addition effectively compensated for the lack of essential amino acids in wheat dough.

## Highlights

• The dough had relatively higher *G'*, *G"* and disulfide bond content at 4 % substitution.
• TBBPEHs or CBBPEHs reduced water absorption and increased formation time of dough.
• TBBPEHs or CBBPEHs at 2–4 % substitution had more continuous gluten network structure.
• TBBPEHs or CBBPEHs addition compensated for specific amino acids in wheat dough.

## Graphical abstract

**Figure:**
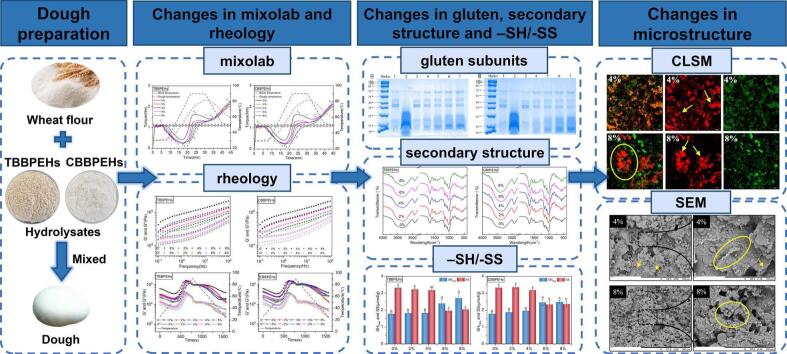

## 1 Introduction

Buckwheat (*Fagopyrum* spp.), a member of the Polygonaceae family, is a significant gluten-free pseudocereal cultivated for its applications in both dietary and medicinal contexts. The milling process of buckwheat generates approximately 25 % buckwheat bran as a by-product. The bran of buckwheat is characterized by substantial concentrations of protein (33–36 %), fat (3–7.4 %), dietary fiber (35–40 %), starch (16–18 %), lipids (9–11 %), and minerals (5–7 %) (Zhou et al., 2022). Therefore, buckwheat bran has considerable potential as a food ingredient. However, its unfavorable flavor has potential to exert a negative influence on the sensory quality of food products. Additionally, the indigestible and non-absorbable properties restricted its utilization in the food industry. The utilization of excellent main functional components in buckwheat to strengthen or improve the processing and nutritional quality of functional foods has become the focus of current research.

Buckwheat protein, as one of the main components of buckwheat bran, has a unique amino acid composition and is particularly rich in eight essential amino acids, including a high proportion of lysine and arginine (Kowalski et al., 2022). It is important to emphasize that lysine is usually the first limiting amino acid in many cereal proteins (Sinkovič et al., 2022), which means that buckwheat protein has great potential to become a valuable supplemental protein source for grains and vegetables. It was found that buckwheat protein decreased cholesterol levels in the liver and plasma and increased fecal excretion of bile acids by modulating the intestinal microbiota in mice (Zhang et al., 2017). Buckwheat protein could also improve glucose tolerance in diabetic patients by increasing the activity of antioxidant enzymes and decreasing the levels of reactive oxygen species such as malondialdehyde (Chiang et al., 2023). Luo et al. (2020) discovered that three peptide fragments (Gly-Glu-Val-Pro-Trp, Tyr-Met-Glu-Asn-Phe, and Ala-Phe-Tyr-Arg-Trp) derived from buckwheat protein through alkaline protease hydrolysis demonstrated stronger antioxidant activity than ascorbic acid. Moreover, Fujimura et al. (2003) successfully isolated two antimicrobial peptides, designated as Fa-AMP 1 and Fa-AMP 2, from buckwheat. Further study by Leung and Ng (2007) revealed that both Fa-AMP 1 and Fa-AMP 2 exhibited significant inhibitory effects on the *fungi Fusarium oxysporum*, as well as various gram-positive and gram-negative bacteria. These above studies have demonstrated that buckwheat proteins and their hydrolysates exhibited a range of biological activities, which appeared to be attributed to the effects of active peptide fragments. Therefore, it is necessary to further investigate whether the functional activities of buckwheat proteins or peptides were still maintained in cereal flour products, as well as to clarify their effects on the processing quality of cereal flour products.

Many studies have demonstrated that the incorporation of protein hydrolysates could change the taste and flavor of wheat flour products, as well as improve their nutritional value by altering the gluten network structure. Guo et al. (2022) reported that the incorporation of soy protein hydrolysates resulted in a decrease in dough stability while enhancing its ductility. These hydrolysates also improved the smoothness and cooking yield of the noodles, with exhibiting the resistance to starch gelatinization and retrogradation. The substitution of 1 or 3 % (w/w) of wheat flour with lima beans and cowpeas hydrolysates significantly enhanced the strength and elasticity of the dough, and also increased the protein content of the bread (Franco-Miranda et al., 2017). Karimi et al. (2021) found that the addition of 4 g/100 g (w/w) of corn germ protein hydrolysates increased the DPPH radical scavenging activity and Fe^2+^ chelating activity of bread by 2.3 times and 2.1 times, respectively, and also improved the texture, hardness and chewiness of the bread during storage. However, studies on the effects of buckwheat proteins or peptides on the processing and functional properties of starch-based foods were still lacking, and clarifying these studies could help in the development of functional cereals-based on buckwheat with improved nutritional qualities through the addition of proteins or peptides.

In this study, Tatary buckwheat bran protein enzymatic hydrolysates (TBBPEHs) and common buckwheat bran protein enzymatic hydrolysates (CBBPEHs) were obtained by treating with pepsin and trypsin. The changes in the thermo-mechanical, rheological, textural, microstructural and molecular properties of non-fermented wheat dough by TBBPEHs and CBBPEHs hydrolysates were investigated, which could lay the foundation for further studies on nutritional cereal-based flour products.

## 2 Materials and methods

### 2.1 Materials

Tartary buckwheat bran (Heifeng No.1) was obtained from Shanxi Yanmen Qinggao Food Co., Ltd. Common buckwheat bran (Yulin Honghua) was obtained from Dingbian County Saixue Grain and Oil Industry and Trade Co., Ltd. Wheat flour was purchased from Xiangnian Foods Co., Ltd. (Nanyang, Henan, China). Pepsin (P7125, E.C.3.4.23.1, ≥400 units/mg protein) and trypsin (T4799, E.C.3.4.21.4, 1000–2000 units/mg protein) were obtained from Sigma-Aldrich Co. (St. Louis, MO, USA). The SDS-PAGE gel assay kit was obtained from Solarbio Co., Ltd. (Beijing, China). Potassium bromide (KBr) was obtained from Tianjin Kemiou Chemical Reagent Co., Ltd. (Tianjin, China). All other reagents were of analytical grade.

### 2.2 Preparation of buckwheat bran protein

The buckwheat bran protein was extracted according to the method described by Yu et al. (2022) with some modifications. Buckwheat bran flour was milled and sieved through an 80-mesh sieve, then mixed with 75 % (v/v) ethanol at a ratio of 1:10 (m/v) and stirred constantly at 250 rpm/min and 25 °C for 20 min to remove pigments. After the above steps were repeated for 5 times, the enriched precipitates were remixed in petroleum ether at a ratio of 1:10 (m/v) for 12 h to remove lipids. The pre-treated buckwheat bran flour was air-dried at 50 °C for 4 h after washing for 3 times with 75 % ethanol (v/v).

The mixture of pre-treated buckwheat bran flour and distilled water at 1:10 (m/v) were adjusted to a pH of 10.0 with 1.0 M NaOH and stirred continuously for 1 h at 40 °C and 200 rpm/min. The supernatant was collected after centrifugation at 3990 ×*g* for 20 min and then adjusted to pH 4.2 with 1.0 M HCl, followed by centrifugation at 3990 ×*g* for 15 min. The enriched precipitate (buckwheat bran protein) was redissolved in distilled water at 1:3 (v/v) and freeze-dried, then stored at 25 °C for further application.

### 2.3 Preparation of enzymatic hydrolysates

A suspension of freeze-dried buckwheat bran protein and distilled water were mixed at a ratio of 1:50 (m/v), and was adjusted to a pH of 2.0 with 1.0 M HCl. Then pepsin was added at a final enzyme-to-substrate ratio of 5 % (E/S) and incubated for 1 h at 37 °C. Subsequently, the mixture was adjusted to pH 8.0 with 1.0 M NaOH, and trypsin was added at the same final enzyme-to-substrate ratio (E/S) with the incubation for 3 h at 37 °C. The mixture was inactivated at 90 °C for 15 min and cooled to room temperature, then centrifuged at 3000 r/min for 10 min (Han et al., 2022). The enzymatic hydrolysates (TBBPEHs and CBBPEHs) were obtained by freeze-drying the supernatant. The chemical composition of all samples was shown in Supplemental Table 2.

### 2.4 Mixolab analysis

The mixtures of wheat flour with TBBPEHs or CBBPEHs were prepared at mass ratios of 100/0, 98/2, 96/4, 94/6 and 92/8 (dry basis). The thermal-mechanical properties of the dough were tested during dough formation, heating gelatinization and cooling using the Mixolab mixed experimental mode of the Chopin+ standard test protocol (Chopin, Tripette et Renaud, Paris, France) according to AACC International approved method 54–60.01 (American Association of Cereal Chemists, 2010). The moisture content of each sample was determined using a moisture content tester before testing. Firstly, the sample was held at 30 °C for 8 min, and heated to 90 °C for 7 min, and finally cooled to 50 °C. The water absorption rate, development time, initial consistency (C1), minimum torque (C2), peak torque during heating (C3), minimum torque during the heating period (C4), torque obtained after cooling at 50 °C (C5) and stability time were calculated.

### 2.5 Dough preparation

The mixtures of wheat flour with TBBPEHs or CBBPEHs were prepared at mass ratios of 100/0, 98/2, 96/4, 94/6 and 92/8 (dry basis), which corresponded to 0, 2, 4, 6 and 8 % (w/w) TBBPEHs or CBBPEHs in relation to the mass of the total mixed flour (100 g) in each sample. An appropriate amount of distilled water (calculated as 92 % water absorption from Mixolab mixing experiment) was added and mixed with the flours for 15 min at 120 rpm/min using a dough mixer (M5, Qingdao, China). Finally, the well-mixed dough was packaged in a sealed bag for 20 min for further application. A mixing duration of 15 min was selected to the facilitate homogeneous distribution of all formulation components, ensuring thorough integration of enzymatic hydrolysates and wheat flour while minimizing compositional inconsistencies.

### 2.6 Rheological properties of dough

The rheological properties of dough were analyzed using a food rheometer (DHR-2, TA Instruments, New Castle, DE, USA) according to the method described by Pei et al. (2024). All the prepared dough samples were equilibrated at room temperature for 10 min and then loaded on a metal plate with a 40 mm diameter and 2000 μm gap at 25 °C. Frequency sweep tests were performed at 0.1–100 Hz and 1 % strain. Temperature sweep tests were monitored at a frequency of 1 Hz and 1 % strain. A temperature curve with a linear rate of 5 °C/min from 25 to 90 °C and 90 to 25 °C was used. The strain parameters were in the linear viscoelastic region according to the strain scanning test. Before each experiment, the edges of the samples were covered with a thin layer of silicone oil to prevent water evaporation.

### 2.7 Texture measurement of dough

The structural properties of all samples were measured using the Texture Analyzer (TA-XT Express, Stable Micro Systems Ltd., UK) by performing a texture profile analysis (TPA) according to the method described by Duan et al. (2024) with minor modifications. All prepared dough samples were equilibrated at room temperature for 10 min. The dough samples (5.0 g) were weighed and placed into metal molds with 1.0 cm diameter to form round pieces. The dough samples were compressed twice to 50 % of their original height using a 36.0 mm diameter cylindrical probe (P/36R). The test speed and trigger force were 1 mm/s and 5 g, respectively. The hardness, springiness, chewiness and cohesiveness were calculated to evaluate the qualities of dough samples. All samples were measured at least six times.

### 2.8 Low field nuclear magnetic resonance

Water distribution and water mobility in the dough were assessed using the LF-NMR instrument (NMI20–015 V-I, Shanghai Niu Mag Electronic Technology Co., Ltd., Shanghai, China) following the method described by Duan et al. (2024). The dough samples (2.0 g) were weighed and placed into NMR tubes with 15.0 cm diameter to form round cylinders, then positioned in the NMR probes. Carr-Purcell-Meiboom-Gill (CPMG) sequences were utilized to determine the spin-spin relaxation time (T_2_) The specific parameters used were: SF = 21 MHz, O1 = 304,765.52 Hz, P1 = 13.00 us, TD = 80,022, TW = 3500.00 ms, P2 = 26.00 ms, TE = 0.200 ms, NECH = 2000, SW = 200 KHz, RFD = 0.08 ms, RG1 = 20.0 db, DGR1 = 3, NS = 16. The same level was measured three times. The T_2_ values of the samples were calculated using inversion software (Niu Mag Ver4.0, Shanghai Niu Mag Electronic Technology Co., Ltd., Shanghai, China). Each measurement was performed in triplicate.

### 2.9 Determination of the free amino acids

The prepared dough samples were freeze-dried and crushed into powder (passed through a 100-mesh sieve). 100 mg of powder sample along with 4.0 mL 6.0 M HCl was mixed and incubated for 15 min in a nitrogen environment. The mixed sample was diluted to 100 mL with distilled water after being hydrolyzed at 110 °C for 24 h. The mixed solution (2 mL) was dried under nitrogen environment at 60 °C and then mixed with an aliquot of 0.02 M HCl. Finally, the free amino acids of pre-treated sample were analyzed using an amino acid automatic analyzer (Biochrom30+, Biochrom Ltd., UK) after filtering through a 0.22 μm filter membrane (Xu et al., 2022).

### 2.10 SDS–PAGE of dough

The freeze-dried dough sample (30 mg) was dissolved in 1 mL distilled water and mixed with sample loading buffer at 3:1 (v/v). The mixture was incubated in boiling water for 5 min and centrifuged at 10,000 ×*g* for 10 min, and then 10 μL of the pre-treated sample was taken for SDS-PAGE electrophoresis (separation gel was 12 %, and concentration gel was 4 %). The electrophoresis was performed in constant-current mode, with 25 mA for the concentrated gel and 35 mA for the separated gel. After electrophoresis, the gel was treated with fixation, coloration and decolorization, and then placed in the gel imaging system (GelDocXR, Bio-Rad Laboratories, USA) to observe the molecular weight change of the dough protein. All reagents used were of analytical reagent grade.

### 2.11 Measurement of the secondary structure of dough

The FT-IR spectrum of dough samples was obtained using a Fourier transform infrared spectrometer (VERTEX70, Bruker Corporation, Karlsruher, Germany) following the method described by Pei et al. (2024). All samples were pressed into conventional KBr (chromatography grade) pellets at a ratio of 1:100 (w/w) and tested in the wavelength range of 4000–400 cm^−1^. All the samples were successively scanned 32 times. Spectral data were processed using Peakfit V4.12 software and each sample was performed in triplicate.

### 2.12 Free sulfhydryl (-SH) and disulfide bond (-SS) content

The content of sulfhydryl and disulfide bonds in the dough was determined using Ellman's colorimetric method described by Liu et al. (2016) with minor modifications. The freeze-dried dough sample (75 mg) was crushed and dissolved in 1 mL 8 M Tris-glycine buffer (including 3 mM EDTA, 0.1 M Glycine and 0.1 M Tris-HCl) at pH 8.0. Subsequently, 4.7 g of guanidine hydrochloride was added and diluted to 10 mL with buffer. As for free SH content, 1 mL of the sample dispersion along with 4 mL of urea-guanidine hydrochloride and 0.05 mL of 5.5′-dithiobis-2-nitrobenzoic acid (DTNB) were mixed and then the absorbance of the mixtures was measured at 412 nm using a UV–vis spectrophotometer (UV-2400, SDPTOP, Shanghai, China). Tris-glycine buffer was used as blank control. As for total SH content, 1 mL of the sample dispersion along with 4 mL of urea-guanidine hydrochloride and 0.05 mL of β-mercaptoethanol were mixed and incubated at 25 °C for 1 h in the dark. Then 10 mL of 12 % trichloroacetic acid was added and incubated for 1 h before the solution was centrifuged at 3990 ×*g* for 10 min. The enriched precipitate was washed twice with trichloroacetic acid and dissolved in 10 mL of 8 M urea and 0.04 mL of DTNB. The absorbance was measured at 412 nm. The free SH and total SH contents were calculated according to the equation below:

(1) $$\mathrm{SH}\left(\mathrm{\mu M}/g\right)=\left(\frac{73.53\times A\times D}{C}\right)\times 100$$

Where A represents the absorbance of the sample at 412 nm after removal of the reagent blank. D and C represent the dilution ratio and sample concentration, respectively.

The disulfide bond (SS) contents were calculated according to the equation below:

(2) $$\mathrm{SS}\left(\mathrm{\mu M}/g\right)=\left(\frac{\mathrm{SH}-{\mathrm{SH}}_{F}}{2}\right)\times 100$$

### 2.13 Confocal laser scanning microscopy (CLSM)

The dough samples were sliced (30 μm thickness) with a with a freezing microtome (Leica CM 1950, Leica Instruments, Germany) after cryo-embedding with tissue freezing medium and then placed on glass slides. All samples were stained with a 1:1 mixture of 0.02 % (w/v) fluorescein 5-isothiocyanate (FITC) in acetone and 0.01 % (w/v) rhodamine B in water (Silva et al., 2013). The CLSM images were captured by a Laser Scanning Confocal Microscope (FV3000, Olympus Corporation, Japan) with a 400 × objective, 1024 × 1024 pixels, and 100 Hz frequency. FITC and Rhodamine B were stimulated by 488 nm and 561 nm lasers, respectively. The emission fluorescence of starch and protein was detected at 500–540 nm and 570–620 nm, respectively.

### 2.14 Scanning electron microscopy (SEM)

The microstructures of freeze-dried dough samples were analyzed using a scanning electron microscope (TM3030Plus, Hitachi Ltd., Tokyo, Japan). The freeze-dried dough samples were divided into small pieces (5 mm × 5 mm) with a blade and attached to the sample holders. All the samples were gold-coated and observed using an electron beam with an acceleration voltage of 20 kV (Li et al., 2022).

### 2.15 Statistical analysis

All results were presented as mean ± standard deviation and repeated at least three times. Statistical analysis was carried out using SPSS Version 26.0 software (IBM software, Chicago, USA). Significance (*P* < 0.05) analysis was performed using Duncan procedure.

## 3 Results and discussion

### 3.1 Thermomechanical properties of mixed flour

**Fig. 1:**
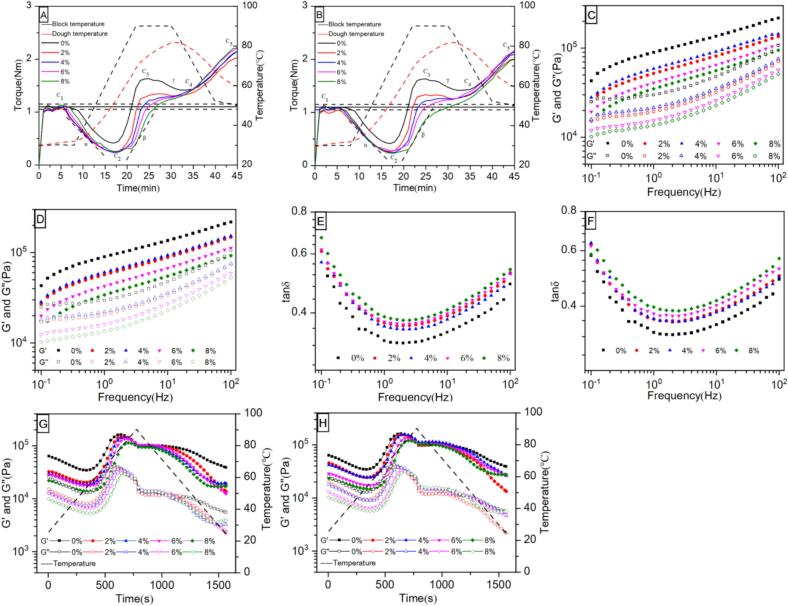
Effects of TBBPEHs (A, C, E, G) and CBBPEHs (B, D, F, H) hydrolysates on the Mixolab (A, B) and rheological properties (C, D, E, F) of wheat flour.

The Mixolab curves as well as statistical data for all the samples were shown in Fig. 1A and B and Table 1. As the amount of hydrolysate substitution increased, the water absorption of flour significantly decreased from 56.00 % to 48.95 % (Table 1), suggesting that some hydrophobic groups in hydrolyzed buckwheat bran protein might be overexposed by the addition of hydrolysates, which led to a decrease in the water absorption of the dough (Yang, Wang, et al., 2024). Compared to wheat dough (1.79 min), the addition of both TBBPEHs and CBBPEHs hydrolysates significantly increased the formation time of the dough samples to 4.85–5.27 min and 4.34–4.99 min, respectively, while the stable time was significantly decreased to 5.83–7.05 min and 6.40–7.10 min, respectively (*P* < 0.05). These changes could be attributed to the interaction between TBBPEHs or CBBPEHs hydrolysates and gluten proteins, which allowed more time for the dough in the initial forming stage. However, as the dough further formed, the TBBPEHs and CBBPEHs hydrolysates disrupted the potential disulfide bonds that could be formed with the gluten proteins, which reduced the stability of the gluten network, resulting in a shorter stable time of the dough.

**Table 1:** Mixolab analysis results of TBBPEHs and CBBPEHs at different content.

Hydrolysates content (%)	Water absorption (%)	Forming time (min)	Stable time (min)	Maximum torque C1 (N·m)	Minimum torque C2 (N·m)	Peak viscosity C3 (N·m)	Trough viscosity C4 (N·m)	Final viscosity C5 (N·m)
Wheat dough	56.00 ± 0.00^Aa^	1.79 ± 0.07^Cc^	8.67 ± 0.12^Aa^	1.13 ± 0.02^Aa^	0.42 ± 0.02^Aa^	1.66 ± 0.02^Aa^	1.46 ± 0.06^Aa^	2.14 ± 0.06^Aa^
TBBPEHs								
2	54.80 ± 0.00^B^	4.85 ± 0.25^B^	5.83 ± 0.06^D^	1.10 ± 0.03^A^	0.27 ± 0.03^B^	1.37 ± 0.03^B^	1.31 ± 0.02^B^	2.02 ± 0.02^B^
4	52.20 ± 0.00^C^	4.83 ± 0.13^B^	5.95 ± 0.07^D^	1.11 ± 0.01^A^	0.24 ± 0.01^B^	1.05 ± 0.03^C^	1.30 ± 0.02^B^	2.15 ± 0.00^A^
6	50.63 ± 0.46^D^	5.07 ± 0.13^AB^	6.47 ± 0.25^C^	1.14 ± 0.01^A^	0.26 ± 0.01^B^	0.80 ± 0.02^D^	1.26 ± 0.01^BC^	2.18 ± 0.03^A^
8	48.95 ± 0.64^E^	5.27 ± 0.09^A^	7.05 ± 0.21^B^	1.11 ± 0.04^A^	0.23 ± 0.03^B^	0.58 ± 0.02^E^	1.21 ± 0.04^C^	2.18 ± 0.05^A^
CBBPEHs								
2	55.20 ± 0.61^b^	4.74 ± 0.23^a^	6.45 ± 0.07^c^	1.07 ± 0.03^a^	0.28 ± 0.02^b^	1.37 ± 0.02^b^	1.28 ± 0.05^b^	2.05 ± 0.12^ab^
4	52.70 ± 0.44^c^	4.34 ± 0.19^b^	6.40 ± 0.00^c^	1.10 ± 0.04^a^	0.25 ± 0.01^b^	1.11 ± 0.03^c^	1.27 ± 0.01^b^	2.16 ± 0.06^a^
6	50.70 ± 0.00^d^	4.99 ± 0.05^a^	6.75 ± 0.21^bc^	1.12 ± 0.02^a^	0.26 ± 0.02^b^	0.87 ± 0.03^d^	1.22 ± 0.03^b^	2.14 ± 0.07^ab^
8	49.03 ± 0.49^e^	4.77 ± 0.02^a^	7.10 ± 0.28^b^	1.10 ± 0.04^a^	0.25 ± 0.03^b^	0.61 ± 0.03^e^	1.09 ± 0.03^c^	2.00 ± 0.07^b^

All values are averages ± SD of three runs. The different letters in the same column are significantly different (*P* < 0.05). The uppercase and lowercase superscripts represent significant differences in TBBPEHs -treated and TBBPEHs -treated doughs, respectively (*P* < 0.05).

The maximum torque (C1) value could reflect the maximum torque during the dough mixing process. There was no significant difference in C1 values between samples at the initial stage, which was due to the addition of different amounts of water to achieve a consistent value of 1.10 ± 0.10 Nm (Xu et al., 2020). The minimum torque (C2) value could indicate the damping effect of the dough on proteins under the combined influence of mechanical stress and temperature. As the temperature increased from 30 °C to 90 °C, the C2 value of the dough decreased significantly to 0.27–0.23 Nm and 0.28–0.25 Nm, respectively, with the increased TBBPEHs and CBBPEHs hydrolysates content, compared to the control group at 0.42 Nm. This demonstrated a significant decrease in both protein content and stability, which might be due to the weakened interactions between hydrolysate and gluten, resulting in an unstable gluten network structure. During the temperature holding stage (90 °C), the peak viscosity (C3) value, which represented the maximum torque during dough formation, decreased significantly from 1.66 Nm to 1.37–0.58 Nm and 1.37–0.61 Nm, respectively, with the increase of TBBPEHs and CBBPEHs hydrolysates. It suggested that the TBBPEHs and CBBPEHs hydrolysates could retard the aging process of the starch in the dough, thus affecting the elastic-viscous properties of the dough. In addition, the trough viscosity (C4) value of the control group was 1.46 Nm, which also decreased significantly to 1.31–1.21 Nm and 1.28–1.09 Nm, respectively, with the addition of TBBPEHs and CBBPEHs hydrolysates, further indicating the influence of hydrolysate on starch gelatinization and properties. Finally, as the temperature decreased from 90 °C to 50 °C, the final viscosity (C5) of the dough increased due to starch retrogradation. This observation aligned with the results of rheological experiments, indicating that the viscosity of the dough changes during cooling due to the rearrangement of starch molecules and the redistribution of water (Cingöz et al., 2023). In summary, the water absorption, formation time, stability time, and the values of C2, C3, C4, and C5 of the dough showed concentration-dependent characteristics.

### 3.2 Rheological properties of dough

The effect of TBBPEHs and CBBPEHs hydrolysates on dough viscoelasticity were evaluated by frequency and temperature scanning of non-fermented wheat doughs. The frequency trends of storage modulus (*G'*) and loss modulus (*G"*) of all dough samples were shown in Fig. 1C and D. Both *G'* and *G"* showed an increasing tendency with the frequency increase. *G'* was greater than *G"* at the same frequency, which demonstrated that all the samples were solid-like behavior, and the dough had an elastic-fluid property. This could be attributed to the fact that when the oscillation frequency increased, the starch granules were embedded in the dough and acted as a supporter, which enhanced the strength of the gluten network structure and provided a strong bonding force (Sivaramakrishnan et al., 2004). However, both *G'* and *G"* were lower in the TBBPEHs and CBBPEHs hydrolysates groups compared to the control group (0 %) and showed a slightly dose-dependent properties (decreased with concentration increasing) when the concentration was greater than 4 %. This might be due to the fact that the TBBPEHs and CBBPEHs hydrolysates could not form a network structure and their presence also interfered with the formation of wheat gluten protein network (Li et al., 2021).

The loss factor, defined as tan *δ* = *G"/G'*, is positively correlated with the degree of molecular polymerization in the system. A decrease in tan *δ* indicates a higher system elastic ratio, reduced fluidity, and a higher degree of polymerization or number of polymers in the system components. Conversely, a high viscosity ratio corresponds to strong fluidity and a high proportion of molecules with low polymerization degrees (Kotsiou et al., 2021). As shown in Fig. 1E and F, the loss factor of all samples was less than 1.0 during the frequency sweep, indicating that the samples were more elastic than viscous. The tan δ showed a trough tendency with the increase of frequency. When the frequency ranged from 0.1 to 1.0 Hz, the gluten network structure in dough was relatively complete, which conferred better elasticity to the dough. As the frequency increased, the internal cross-linking of gluten protein weakened and the fluidity of the gel system of the dough increased. The tan δ of both TBBPEHs and CBBPEHs hydrolysates (2–8 %) was higher than that of the blank group (0 %), which further suggested that the presence of hydrolysates affected the formation of wheat gluten network structure, and resulted in weakening of dough viscoelastic properties. These results were consistent with the finding of Li et al. (2021) that the addition of hydrolyzed soy protein could weak the gluten protein structure and thus affect the rheological properties. Furthermore, it was worth noting that the tan δ values of the 4 % TBBPEHs and CBBPEHs hydrolysates group were lower than those of the 2 %, 6 % and 8 % groups after frequency greater than 2.0 Hz, which demonstrated that the 4 % TBBPEHs and CBBPEHs hydrolysates had less effect on the viscoelastic properties of the dough.

The effect of TBBPEHs and CBBPEHs on the thermodynamic properties of dough was shown in Fig. 1G and H. Both *G'* and *G"* of all samples showed a similar tendency, which initially decreased, then increased, and subsequently decreased again after reaching the peak values as the temperature increased. During the initial heating stage (25–60 °C), the dough began to gradually soften and was accompanied by a decrease in *G'* and *G"*. Both TBBPEHs and CBBPEHs hydrolysates intensified this decreasing tendency. This might be due to the non-covalent bonding between enzymatic hydrolysates and the starch, protein or other components in wheat dough as well as their sensitivity to temperature, which restricted the interactions within the dough and caused a decrease in *G'* and *G"* after heating (Zhang et al., 2022). As the temperature increased (60–80 °C), proteins began to be denatured, and starch granules further disintegrated. The heat-treatment induced the formation of a continuous multiphase gel system, which promoted the denaturation of the dough and contributed to a significant increase in *G'*, *G"* and viscoelastic properties. However, as the temperature increased to the range of 78 to 84 °C, both *G'* and *G"* of all samples reached their peaks, and the peak values of the doughs gradually decreased with the addition of TBBPEHs (Fig. 1G), corresponding to a gradually increase for the peak temperatures. Similar results were also found in CBBPEHs-treated doughs (Fig. 1H). The reduction of *G'* and *G"* values suggested that heat-treatment significantly altered the rheological properties of the dough, leading to softening and disintegration of the gluten network structure rather than the formation of a rigid frames. The increased peak temperature indicated that the addition of hydrolysates could enhance the thermal stability of the dough.

### 3.3 Textural characteristics of dough

The effects of varying substitution levels of TBBPEHs and CBBPEHs hydrolysates on the quality and textural properties of dough were shown in Table 2. Compared to that of wheat dough (0 %), the presence of TBBPEHs and CBBPEHs hydrolysates resulted in a significant decrease (*P* < 0.05) in the hardness and chewiness of the dough samples to the ranges of 115.77–166.30 and 94.66–121.89, respectively, but the cohesiveness was significantly increased (*P* < 0.05). These results indicated that different concentrations of TBBPEHs and CBBPEHs hydrolysates could disrupt the structure of the protein-starch network thus significantly altering the textural properties of the dough (Jia et al., 2019). The TBBPEHs-treated dough samples at 4 % substitution rate showed higher springiness (*P* < 0.05) compared to other dough samples in the TBBPEHs groups, while this result was not found in CBBPEHs groups. This finding suggested that the 4 % TBBPEHs-treated dough might have better potential processing properties in the preparation of weak or medium gluten products. However, the differences in the textural properties of the doughs in TBBPEHs and CBBPEHs hydrolysates groups could be attributed to differences in protein sources, degree of hydrolysis, molecular weight sizes, viscosities, or other properties of the enzymatic hydrolysates (Guo et al., 2022).

**Table 2:** Texture and water distribution properties of dough.

Polypeptides content (%)	Hardness (g)	Springiness	Chewiness	Cohesiveness	T_21_ (ms)	T_22_ (ms)	T_23_ (ms)	A_21_ (%)	A_22_ (%)	A_23_ (%)
Wheat dough	230.36 ± 4.34^Aa^	0.93 ± 0.00^Ba^	158.47 ± 3.34^Aa^	0.73 ± 0.00^Be^	0.18 ± 0.02^Cc^	10.72 ± 0.00^Aa^	75.65 ± 0.00^Cb^	8.66 ± 0.26^Aa^	87.72 ± 0.34^Dc^	3.61 ± 0.10^Aa^
TBBPEHs										
2	148.42 ± 7.15^B^	0.93 ± 0.00^B^	118.61 ± 4.48^B^	0.86 ± 0.03^A^	0.21 ± 0.03^BC^	12.40 ± 0.10^A^	86.98 ± 0.00^B^	8.40 ± 0.02^A^	87.70 ± 0.23^D^	3.47 ± 0.01^B^
4	149.38 ± 2.48^BC^	0.94 ± 0.00^A^	121.89 ± 3.31^B^	0.87 ± 0.01^A^	0.23 ± 0.01^B^	11.89 ± 1.63^A^	86.98 ± 0.00^B^	5.19 ± 0.06^D^	91.63 ± 0.07^A^	3.54 ± 0.04^AB^
6	135.85 ± 7.04^BC^	0.92 ± 0.00^C^	109.18 ± 5.86^BC^	0.87 ± 0.01^A^	0.24 ± 0.01^B^	11.53 ± 1.13^A^	100.00 ± 0.00^A^	7.75 ± 0.01^B^	88.43 ± 0.42^C^	3.55 ± 0.02^AB^
8	123.77 ± 8.86^C^	0.91 ± 0.00^D^	95.45 ± 8.63^C^	0.84 ± 0.06^A^	0.33 ± 0.00^A^	11.78 ± 0.65^A^	100.00 ± 0.00^A^	7.11 ± 0.00^C^	89.18 ± 0.08^B^	3.48 ± 0.01^B^
CBBPEHs										
2	166.30 ± 10.07^b^	0.93 ± 0.00^a^	122.47 ± 3.34^b^	0.79 ± 0.02^d^	0.19 ± 0.00^bc^	10.72 ± 0.00^a^	75.65 ± 0.00^b^	8.26 ± 0.07^b^	88.49 ± 0.32^c^	3.26 ± 0.25^a^
4	149.06 ± 6.91^c^	0.93 ± 0.01^a^	113.93 ± 3.86^bc^	0.82 ± 0.01^c^	0.23 ± 0.01^b^	10.72 ± 0.00^a^	75.65 ± 0.00^b^	4.87 ± 0.14^e^	91.58 ± 0.48^a^	3.55 ± 0.34^a^
6	137.99 ± 6.09^c^	0.93 ± 0.00^a^	110.09 ± 5.71^c^	0.86 ± 0.02^b^	0.28 ± 0.01^a^	10.72 ± 0.00^a^	75.65 ± 0.00^b^	6.29 ± 0.03^d^	90.17 ± 0.38^b^	3.54 ± 0.41^a^
8	115.77 ± 4.29^d^	0.92 ± 0.00^b^	94.66 ± 3.89^d^	0.89 ± 0.01^a^	0.31 ± 0.02^a^	10.72 ± 0.00^a^	86.98 ± 0.00^a^	7.04 ± 0.04^c^	89.66 ± 0.16^b^	3.30 ± 0.20^a^

All values are averages ± SD of three runs. The different letters in the same column are significantly different (*P* < 0.05). The uppercase and lowercase superscripts represent significant differences in TBBPEHs -treated and TBBPEHs -treated doughs, respectively (*P* < 0.05).

### 3.4 Water distribution and migration of dough

The T_2_ inversion diagram of dough moisture obtained using the CPMG-T_2_ pulse sequence and fitting analysis (Supplemental Fig. 1). Each curve displayed three peaks, indicating the existence of at least three distinct moisture states within the dough. The time constants and area percentages for each peak were labeled as T_21_ (0.01 to 1 ms), T_22_ (1 to 40 ms), and T_23_ (>40 ms), with corresponding areas denoted as A_21_, A_22_, and A_23_. These peaks correspond to tightly bound water, weakly bound water, and free water, respectively. The addition of TBBPEHs and CBBPEHs hydrolysates significantly impacted the quantities of tightly bound and free water, particularly at concentrations greater than 4 %.

The effects of TBBPEHs and CBBPEHs hydrolysates on dough relaxation time (T_2_) and the corresponding peak area percentage (A_2_) are detailed in Table 2. Compared to wheat dough (0.18 ms and 75.65 ms), TBBPEHs-treated doughs showed an increase in T_21_ (0.20 to 0.33 ms) and T_23_ (86.98 to 100.00 ms), but neither of them exhibited dose-dependent properties. Similarly, the CBBPEHs-treated doughs showed an increase in T_21_ and T_23_ from 0.27 and 75.65 ms to 0.33 and 86.98 ms, respectively. These results suggested that enzymatic hydrolysates enhanced the water fluidity by replacing starch and gluten proteins, thereby altering the development of the gluten network and facilitating the conversion of bound water into a more free-flowing state (Dekkers et al., 2018). Furthermore, the addition of hydrolysates resulted in a significant change from tightly bound water (A_21_) to weakly bound water (A_22_). Notably, at a 4 % hydrolysates concentration, the relative content of tightly bound water (A_21_) decreased from 8.66 % to 5.19 % and 4.87 %. In contrast, the relative content of weakly bound water (A_22_) increased from 87.72 % to 91.63 % and 91.58 %. There was no significant change observed in the free water (A_23_). Ding et al. (2015) found that peptides can influence the amounts of bound and fixed the water by interacting with water molecules through hydrogen bonding, thereby reducing water binding to the gluten network and enhancing water flow. Consequently, the presence of TBBPEHs and CBBPEHs hydrolysates facilitated the transition of tightly bound water to weakly bound water, leading to a decrease in tightly bound water content in the dough.

### 3.5 Free amino acids of dough

Since amino acid content and composition were the key factors in determining the nutritional and processing quality of the dough (Liang et al., 2022). The composition and content of amino acids in wheat dough, 8 % TBBPEHs dough, 8 % CBBPEHs dough, TBBPEHs and CBBPEHs were characterized (Supplemental Table 1). All samples contained the 17 amino acids used for identification, which included 7 essential amino acids (Thr, Val, Met, Ile, Leu, Phe and Lys) and 10 non-essential amino acids (Asp, Ser, Glu, Gly, Ala, Cys, Tyr, His, Arg and Pro). Both TBBPEHs and CBBPEHs hydrolysates were particularly enriched in essential amino acids, with notable high levels of Thr (28.49 and 30.39 mg/g), Val (37.19 and 33.16 mg/g), Met (16.70 and 14.58 mg/g), Ile (28.36 and 24.88 mg/g), Leu (50.81 and 48.38 mg/g), Phe (54.05 and 51.71 mg/g) and Lys (56.46 and 56.66 mg/g). Compared to wheat dough, the incorporation of TBBPEHs and CBBPEHs hydrolysates resulted in the increased levels of Thr (2.32 mg/g and 1.79 mg/g), Val (3.46 mg/g and 3.13 mg/g), Met (1.15 mg/g and 1.03 mg/g), Ile (1.72 mg/g and 1.26 mg/g), Leu (3.92 mg/g and 3.18 mg/g), Phe (3.45 mg/g and 3.71 mg/g), and Lys (5.04 mg/g and 4.51 mg/g) in the dough. Notably, TBBPEHs exhibited a greater enhancement capacity in increasing these essential amino acids compared to CBBPEHs. Furthermore, the total essential amino acid content in TBBPEHs dough (8 %) increased by 31.00 %, while CBBPEHs dough (8 %) showed an increase of 17.31 %. It has been reported that lysine and threonine were the first and second limiting amino acids in food, respectively, playing crucial roles in protein synthesis, energy metabolism, and mineral absorption (Shruti Shukla et al., 2023). Therefore, the addition of highly active enzymatic products of buckwheat bran proteins compensated for the lack of the amounts or types of lysine and threonine in wheat flour.

### 3.6 Change of gluten subunits induced by TBBPEHs and CBBPEHs addition

**Fig. 2:**
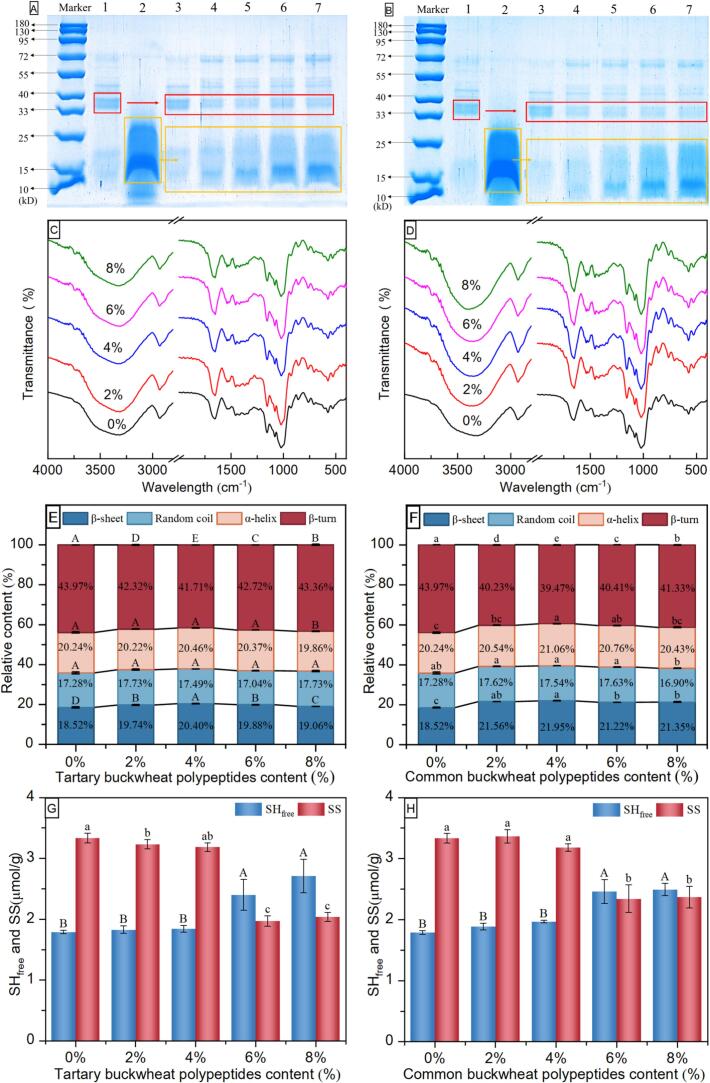
Changes in SDS-PAGE (A, B), FTIR spectra (C, D), secondary structure relative content (E, F) and –SH / –SS content (G, H) of doughs in the presence of TBBPEHs (A, C, E, G) and CBBPEHs (B, D, F, H) hydrolysates. M represented Marker. Lanes 1 to 7 represented wheat flour, TBBPEHs or CBBPEHs, 0 %, 2 %, 4 %, 6 %, and 8 % TBBPEHs or CBBPEHs dough, respectively. The uppercase and lowercase letters represent significant differences in –SH and –SS contents, respectively (*P* < 0.05).

In order to investigate whether TBBPEHs and CBBPEHs hydrolysates induced cross-linking between gluten proteins and affected the interaction between hydrolysates and gluten proteins, the molecular weight distribution of gluten proteins was analyzed using SDS-PAGE (Fig. 2A and B). The results showed that wheat protein predominantly dispersed within the 15–72 kDa range (Band 1). Specifically, molecular weights between 10 and 25 kDa are classified as gliadin, those ranging from 33 to 55 kDa are identified as low molecular weight glutenin subunits (LMS), and those ranging from 55 to 72 kDa are recognized as high molecular weight glutenin subunits (HMS) (Lou et al., 2023). In Band 2, the molecular weight of buckwheat bran protein after enzymatic hydrolysis was observed to range from 10 to 25 kDa. All samples containing hydrolysates displayed typical molecular weight bands associated with wheat dough. It was important to highlight that as the concentration of hydrolysates rises, the distinctive bands associated with wheat protein progressively diminished, evidenced by the absence of protein molecular weights in the range of 33–40 kDa (bands 3 to 7). When the concentration of hydrolysates exceeded 4 %, a prominent band distinctively appeared within the 10–25 kDa range, corresponding to the hydrolysate itself. However, no new molecular weight entities were observed, indicating that no novel structures were formed.

### 3.7 Secondary structure of dough

The location and form of the absorption peaks corresponding to characteristic functional groups in each dough sample were comparable within the 4000–400 cm^−1^ range. Fig. 2C and D showed that the introduction of TBBPEHs or CBBPEHs had no effect on the number of absorption peaks in the gluten FTIR spectra. This suggested that TBBPEHs and CBBPEHs hydrolysates did not alter the specific functional groups present in gluten. For wheat flour samples, the O—H stretching vibration peak appeared around 3400 cm^−1^, while the C—H stretching vibration peak was observed at approximately 2930 cm^−1^. Overall, with the addition of hydrolysates, the absorption peak intensity at 3400 cm-1 associated with hydroxyl O—H increased, indicating the enhanced hydrogen bonding. This increase may result from the exposure of more hydroxyl groups following protein hydrolysis, suggesting that hydrolysates can induce structural rearrangements in gluten by enhancing the number and strength of hydrogen bonds within the dough system. Fig. 2E and F illustrated the effects of enzyme hydrolysates on the β-sheets (1610–1640 cm^−1^), random curls (1640–1650 cm^−1^), α-helices (1650–1660 cm^−1^) and β-turns (1660–1700 cm^−1^) presented in gluten proteins. β-sheets and α-helices were considered as stable and ordered secondary structures, whereas β-turns and random coils were considered disordered structures. As shown in Fig. 2E and F, the relative content of the secondary structure of gluten protein in the dough was: β-turns > α-helixes > β-sheets > Random curls. Compared to the control group (0 %), the dough containing hydrolysates exhibited lower relative contents of β-turns, alongside higher relative contents of β-sheets and α-helices. Compared to the control group, the sum of ordered structures (α-helixes and β-sheets) can increase the maximum amount by 10.96 %, indicating that the addition of hydrolysate could promote the transformation of disordered gluten protein structures into more ordered structures in dough and enhance the elasticity and density of the dough. When the hydrolysate substitution amount was 4 %, the β-sheets and α-helices content of gluten was the highest. Research has shown that both β-sheets and α-helices required substantial hydrogen bonding to maintain stability, with α-helices being the most conformationally stable secondary structure of gluten. The observed increase in both β-sheets and α-helices indicated the enhanced intermolecular hydrogen bonding interactions between gluten molecules, which contributed to improved texture properties of the dough, particularly in terms of elasticity (Chen et al., 2021). This finding was consistent with the SDS-PAGE results, which further confirmed that the binding of hydrolysates occurred through non-covalent interactions.

### 3.8 Changes in free sulfhydryl (-SH) contents and disulfid (-SS) of dough

The contents of –SH and –SS bonds in doughs with varying substitution levels of TBBPEHs and CBBPEHs were presented in Fig. 2G and H. As the substitution levels of TBBPEHs and CBBPEHs hydrolysates increased, the –SH content also rose, while the –SS content exhibited an opposite trend. In the control group, the –SH content was the lowest at 1.79 μmol/g, whereas the –SS content was the highest at 3.33 μmol/g. When the hydrolysate substitution was from 2 % to 4 %, the –SH and –SS contents of the doughs were not differed significantly from those of the control group (0 %), and the trends of TBBPEHs and CBBPEHs hydrolysates were similar. When the substitution amount increased to 6 % ∼ 8 %, the –SH content increased significantly from 1.79 μmol/g to 2.71 μmol/g and 2.49 μmol/g *(P* < 0.05*)*. Conversely, the –SS content decreased significantly from 3.33 μmol/g to 2.04 μmol/g and 2.37 μmol/g, respectively. The –SH bonds content increased and the –SS bonds content reduced in dough, indicating that –SS bonds breakage increased –SH content. Theoretically, the oxidation of SH groups and rapid exchange of –SH and –SS positions contributed to the formation of gluten network structure, which maintained the spatial stability of peptide chains (Zeng et al., 2021). However, our findings suggested that when hydrolysate addition exceeded over 4 %, the –SS content was significantly decreased, indicating insufficient development of the gluten network structure during dough formation, which may negatively impact dough quality. This issue may arise from the interaction between hydrolysates and gluten proteins, which could hinder the formation of the gluten network and disrupt the original gluten structure stabilized by disulfide bonds (Peng et al., 2021). Additionally, the increase in cysteine residues within low molecular weight hydrolyzed peptides may lead to a higher concentration of free sulfhydryl groups (Guo et al., 2018). In summary, the optimal substitution level for TBBPEHs or CBBPEHs was found to be 4 %. At this level, the network structure of the dough remained relatively stable, resulting in favorable physicochemical and processing properties. These findings were consistent with the results obtained from rheological and texture analyses.

### 3.9 CLSM characterization analysis of gluten network

**Fig. 3:**
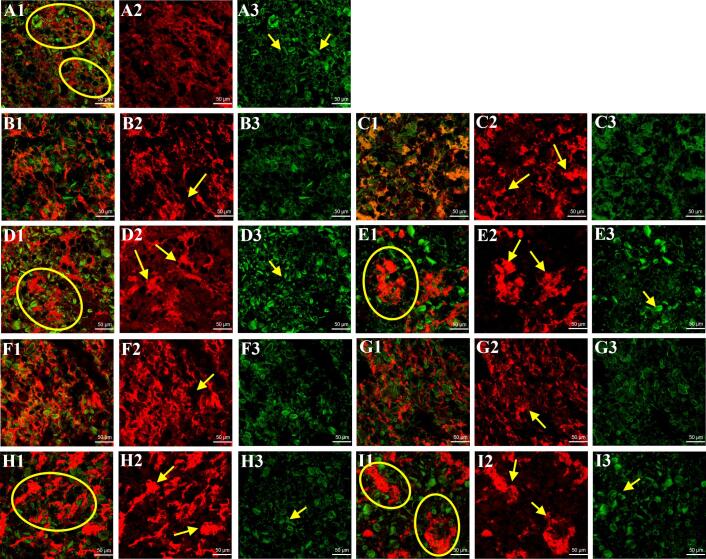
CLSM images of wheat dough (A1, A2 and A3), TBBPEHs-treated dough (from B1 to E3) and CBBPEHs-treated dough (from F1 to I3). The substitution amount of TBBPEHs or CBBPEHs was 0 % (A1, A2 and A3), 2 % (B1, B2, B3, F1, F2 and F3), 4 % (C1, C2, C3, G1, G2 and G3), 6 % (D1, D2, D3, H1, H2 and H3) and 8 % (E1, E2, E3, I1, I2 and I3), respectively. In these images, gluten proteins and starch granules were red and green, respectively. (For interpretation of the references to colour in this figure legend, the reader is referred to the web version of this article.)

To observe the distribution of TBBPEHs and CBBPEHs hydrolysates in the dough, the confocal laser scanning microscopy (CLSM) images of the gluten network were obtained (Fig. 3). In these images, gluten was stained red using Rhodamine B, while starch was stained green with FITC. The complex matrix formed by the interaction of protein and starch was represented in yellow. In wheat dough, a homogeneous and continuous gluten network structure was observed, with starch granules uniformly embedded in the gluten network, forming a continuous mixed gluten-starch matrix (Fig. 3A). For the TBBPEHs-treated samples, a slight aggregation of the gluten network was observed with increasing levels of TBBPEHs (2–4 %), but did not significantly affect the homogeneous distribution of the gluten network. However, at higher concentrations of TBBPEHs (6–8 %), a significant aggregation of the gluten network was observed, and the dough displayed larger gaps and irregularities, accompanied by the appearance of phase separation (Fig. 3E). For the CBBPEHs-treated samples, similar results of phase separation of the dough network structure due to high concentrations (6–8 %) of CBBPEHs were also observed.

Since buckwheat protein hydrolysates (TBBPEHs and CBBPEHs) could exhibit better fluidity or solubility after enzymatic hydrolysis, TBBPEHs and CBBPEHs hydrolysates could be electrostatically attached to the surface of starch granules or wheat gluten proteins when the addition amount was at 2–4 %, which contributed to the network structure of the dough with better dispersibility (Yang, Zhang, et al., 2024). However, when the amount of TBBPEHs or CBBPEHs hydrolysates was increased (6–8 %), large amounts of TBBPEHs or CBBPEHs hydrolysates could induce entanglement or stacking of wheat gluten proteins, which resulted in aggregation of wheat proteins and led to phase separation (Li et al., 2021). Notably, it appeared that the alteration of the dough network structure by CBBPEHs hydrolysates was much weaker and the distribution between the protein and starch phases was more homogeneous at addition levels of 2–4 %. This was attributed to the better viscosity of the CBBPEHs compared to the TBBPEHs (Zhang et al., 2022). These findings indicated that the addition of both TBBPEHs and CBBPEHs hydrolysates altered the gluten network structure of the mixed gluten-starch matrix in wheat dough, leading to larger gaps and increased irregularity that interfered with the self-organization of gluten proteins. (Zhang et al., 2020).

### 3.10 SEM characterization analysis

**Fig. 4:**
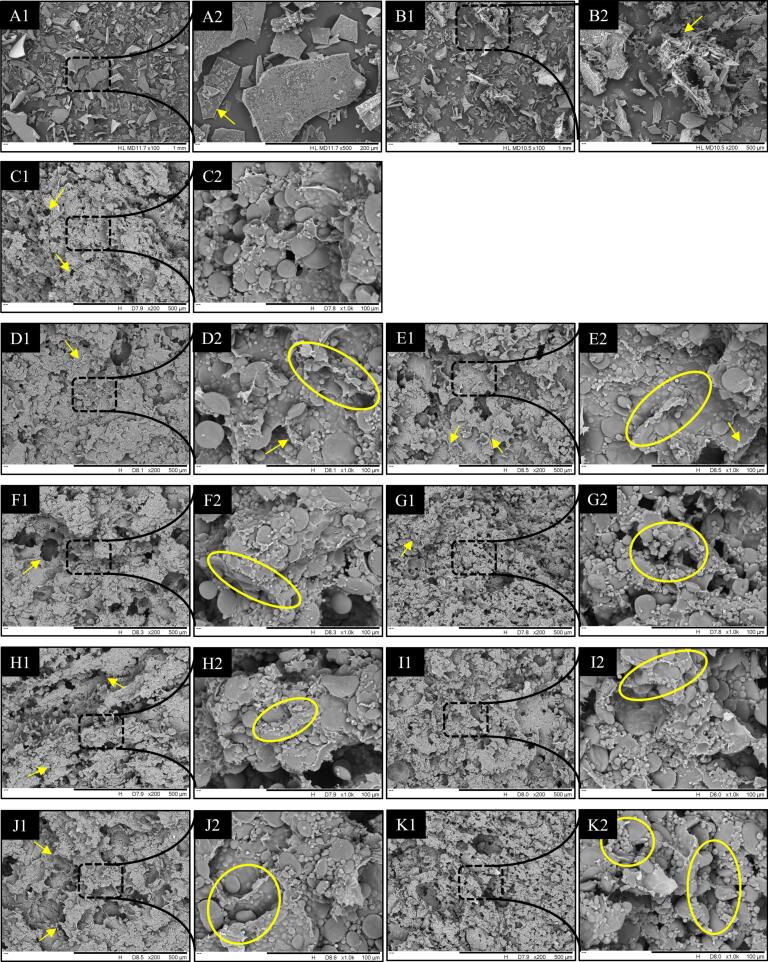
SEM images of TBBPEHs (A1, A2), CBBPEHs (B1, B2), wheat dough (C1, C2), TBBPEHs-treated (from D1 to G2) dough and CBBPEHs-treated (from H1 to K2) dough samples. The substitution amount of TBBPEHs or CBBPEHs was 2 % (D1, D2, H1 and H2), 4 % (E1, E2, I1 and I2), 6 % (F1, F2, J1 and J2) and 8 % (G1, G2, K1 and K2), respectively.

The microstructure of the hydrolysate and dough was examined using scanning electron microscopy (SEM) at various magnifications (100×, 200×, 500×, and 1000×), as depicted in Fig. 4. The freeze-dried TBBPEHs and CBBPEHs hydrolysates exhibited sheet-like rough particles or aggregates with significant variations in particle size (Fig. 4A and B). The SEM of wheat dough showed a homogeneous gluten network structure with uniform pores (Fig. 4C), and the gluten proteins formed a compact network which contained starch granules of various sizes, resulting in a homogeneous and smooth surface.

The addition of 2–4 % TBBPEHs and CBBPEHs hydrolysates led to a more homogeneous structure and a more consecutive gluten network, with starch granules firmly embedded within that network, especially at the 4 % substitution level (Fig. 4E and I). Theoretically, the continuous protein network structure tends to be accompanied by an increase in dough quality. However, the opposite phenomenon was observed in this study. This might be attributed to the fact that the addition of TBBPEHs and CBBPEHs aggregated or both induced wheat protein aggregation, which reduced the network extensibility and led to a decrease in dough stiffness and elasticity. As the hydrolysate substitution reached 6–8 %, the structure of gluten network became discontinuous and incomplete, characterized by larger cavities and increased fractures (Peng et al., 2021). This observation aligned with the findings from the confocal laser scanning microscopy (CLSM). The disruption in the gluten network was attributed to protein aggregation, which hindered the formation of a uniform structural network and left many starch particles exposed, ultimately resulting in a compromised gluten network structure within the dough. Maintaining an optimal level of hydrolysates (4 %) in the dough could facilitate the formation of cohesive network structures. However, when the amount of hydrolysate exceeded this optimal level, it could negatively affect network development. This phenomenon might be attributed to the fact that higher levels of hydrolysates would compete with gluten proteins for available water molecules, and thus hinder the hydration process of gluten, which ultimately destroyed the gluten network and altered the properties of the dough (Gao et al., 2020).

## 4 Conclusions

The present study has shown that substitution with buckwheat bran protein hydrolysates (TBBPEHs and CBBPEHs) in wheat flour resulted in a decrease in dough water absorption, *G'*, *G“*, hardness and chewiness, as well as an increase in dough formation time. However, the complex dough exhibited higher *G'*, *G"* and relative content of disulfide bonds at the 4 % substitution level. SDS-PAGE and FT-IR analyses confirmed that the addition of hydrolysates did not change the molecular weight of the proteins and induce new characteristic bands or absorption peaks in the dough. SEM and CLSM observations revealed that low levels of TBBPEHs and CBBPEHs (2–4 %) promoted a more homogeneous and continuous gluten network, particularly at 4 %, while excessive hydrolysates (6–8 %) negatively affected the gluten network microstructure. Significantly, the amino acid profile of buckwheat bran protein hydrolysates effectively complemented the specific amino acid deficiencies in wheat dough. This finding provided guidance to promote the development of health-focused flour products.

## CRediT authorship contribution statement

**Libo Wang:** Writing – original draft, Software, Project administration, Methodology, Investigation, Funding acquisition, Conceptualization. **Minghuan Yan:** Writing – review & editing, Validation, Software, Formal analysis. **Qiaoming Jiang:** Validation, Formal analysis. **Bing Wang:** Software, Methodology. **Denglin Luo:** Supervision, Resources. **Chonghui Yue:** Visualization, Software. **Jinying Guo:** Resources, Formal analysis. **Ju Qiu:** Supervision, Resources, Project administration, Funding acquisition. **Haoran Wang:** Writing – review & editing, Methodology. **Weijing Wu:** Investigation, Conceptualization. **Yilin Huang:** Writing – review & editing, Supervision, Resources, Project administration.

## Declaration of competing interest

The authors declare that they have no known competing financial interests or personal relationships that could have appeared to influence the work reported in this paper.

## Data Availability

Data will be made available on request.

## Appendix A Supplementary data

Supplementary material Image 1
