# Supplementary material for: Effect of buckwheat bran protein enzymatic hydrolysates on the rheological, textural and structural properties of non-fermented wheat dough
Source: Food Chem X. 2025 Apr 28;27:102501. doi: 10.1016/j.fochx.2025.102501 (PMC12084503; doi:10.1016/j.fochx.2025.102501)
Supplement: Supplementary file 1 — Supplementary material [file mmc1.docx]

Supplementary Fig. 1. Moisture fitting inversion curve of dough.


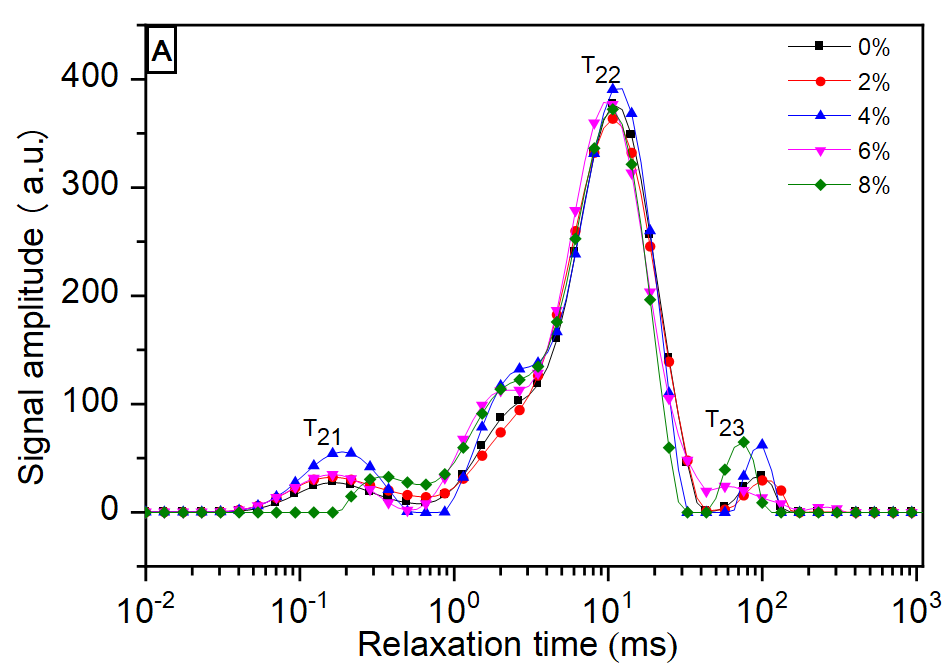

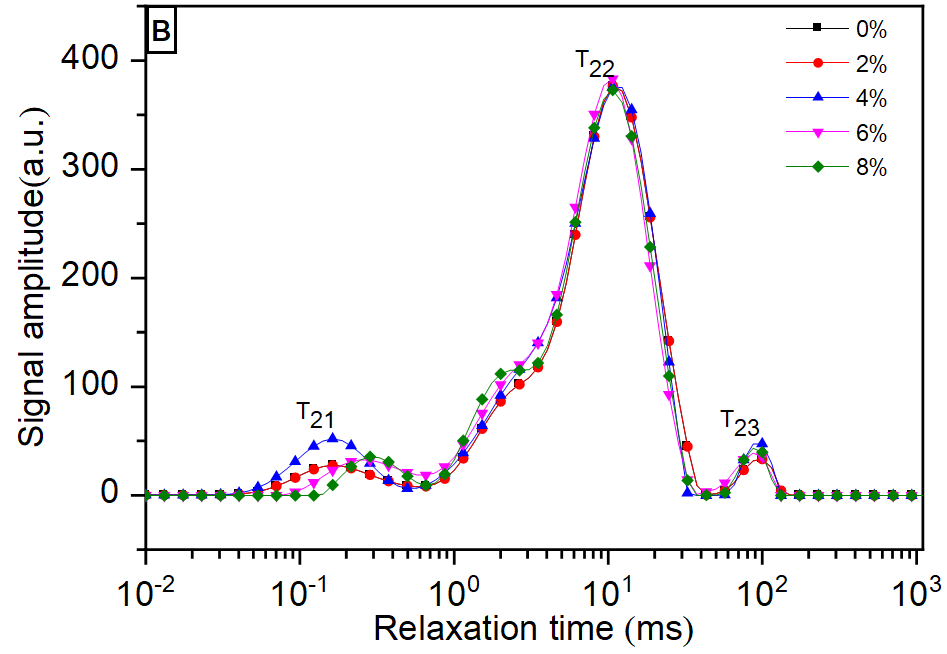


(A) Moisture fitting inversion curve of TBBPEHs dough; (B): Moisture fitting inversion curve of CBBPEHs dough.

Supplementary Table 1. Amino acid component of doughs.

| Amount (mg/100 g) dry basis | | | | | |
| --- | --- | --- | --- | --- | --- |
|  | TBBPEHs | CBBPEHs | Wheat dough | TBBPEHs dough (8%) | CBBPEHs dough (8%) |
| Asp | 94.43 | 91.08 | 8.64 | 17.61 | 17.01 |
| Thr* | 28.49 | 30.39 | 6.07 | 8.39 | 7.86 |
| Ser | 52.33 | 50.28 | 8.98 | 13.46 | 13.47 |
| Glu | 170.47 | 160.49 | 50.89 | 63.50 | 61.04 |
| Gly | 45.22 | 43.37 | 6.66 | 10.60 | 10.35 |
| Ala | 21.97 | 19.85 | 3.79 | 5.69 | 5.36 |
| Cys | 25.90 | 26.81 | 14.39 | 14.44 | 15.17 |
| Val* | 37.19 | 33.16 | 7.45 | 10.91 | 10.58 |
| Met* | 16.70 | 14.58 | 4.21 | 5.36 | 5.24 |
| Ile* | 28.36 | 24.88 | 5.30 | 7.02 | 6.56 |
| Leu* | 50.81 | 48.38 | 11.62 | 15.54 | 14.8 |
| Tyr | 35.37 | 33.52 | 9.96 | 12.63 | 11.33 |
| Phe* | 54.05 | 51.71 | 10.34 | 13.79 | 14.05 |
| His | 35.02 | 29.24 | 6.75 | 9.34 | 8.04 |
| Lys* | 56.46 | 56.66 | 5.54 | 10.58 | 10.05 |
| Arg | 122.86 | 115.76 | 7.08 | 18.43 | 16.83 |
| Pro | 32.93 | 31.64 | 19.69 | 21.74 | 18.89 |
| TAA | 908.54 | 861.80 | 187.35 | 259.02 | 246.61 |
| Note: * indicates essential amino acids | | | | | |

Supplementary Table 2. The chemical compositions of TBBPEHs, CBBPEHs, and wheat flour.

| Sample | Moisture (%) | Protein (%) | Fat (%) | Ash (%) | Starch (%) |
| --- | --- | --- | --- | --- | --- |
| wheat flour | 11.40 ± 0.41^a^ | 16.34 ± 0.24^b^ | 2.10 ± 0.11^a^ | 0.51 ± 0.01^b^ | 68.51 ± 1.25 |
| TBBPEHs | 6.58 ± 0.10^b^ | 85.33 ± 0.95^a^ | 0.51 ± 0.02^b^ | 4.06 ± 0.03^a^ | / |
| CBBPEHs | 6.47 ± 0.24^b^ | 84.04 ± 0.19^a^ | 0.50 ± 0.01^b^ | 4.20 ± 0.11^a^ | / |

Results are expressed in g/100 g sample. The different letters in the same column are significantly different (*P* < 0.05). All values are averages ± SD of three runs. “/” represents the starch is not detected.
